# Cost Effectiveness of Insulin Glargine versus Neutral Protamin Hagedorn Insulin in the Treatment of Type 2 Diabetes Patients in Turkey

**DOI:** 10.36469/9858

**Published:** 2013-08-15

**Authors:** Ilhan Satman, Hayley Bennett, Candeger Yilmaz, Sazi Imamoglu, Goksun Ayvaz, Abdurrahman Comlekci, Demet Ozkaya, Toros Sahin

**Affiliations:** 1 Division of Endocrinology, Faculty of Internal Medicine Istanbul University, Istanbul, Turkey; 2 Swansea Centre for Health Economics Swansea University, Swansea, UK; 3 Division of Endocrinology, Faculty of Internal Medicine Ege University, Izmir, Turkey; 4 Division of Endocrinology, Faculty of Internal Medicine Uludag University, Bursa, Turkey; 5 Division of Endocrinology, Faculty of Internal Medicine Gazi University, Ankara, Turkey; 6 Division of Endocrinology, Faculty of Indernal Medicine Dokuz Eylul University, Izmir, Turkey; 7 Sanofi Turkey, Istanbul, Turkey

**Keywords:** turkey, type 2 diabetes, nph insulin, insulin glargine, cost effectiveness

## Abstract

**Background:** Type 2 diabetes mellitus (T2DM) poses a significant burden on population well being and healthcare expenditure in Turkey, with disease prevalence continuing to increase. Insulin treatment is necessary for patients failing to achieve glycaemic control with lifestyle modification or oral antidiabetic drugs. While neutral protamin Hagedorn (NPH) insulin has been traditionally prescribed for insulin introduction, insulin glargine has been shown to reduce glycated hemoglobin (HbA1c) with a more favourable hypoglycaemic profile.

**Objective:** To evaluate the cost-effectiveness of insulin glargine compared to NPH insulin in patients with T2DM in Turkey, from a Social Security Institution perspective.

**Methods:** A previously published discrete event simulation model of T2DM progression was utilised to characterise the cost-effectiveness of insulin glargine in a Turkish population given the benefits observed in clinical practice. Improvements in glycaemic control have been incorporated using data from The Health Improvement Network (THIN) database in the United Kingdom, combined with meta-regression results describing the relationship between hypoglycaemia and glycaemic control. Outcomes were evaluated over a 40-year horizon, and costs and benefits discounted at an annual rate of 3.5%. Results are reported in Turksih lira (TL), 2012.

**Results:** Over a lifetime, the Incremental Cost-effectiveness Ratio (ICER) of insulin glargine compared to NPH was 40,101 TL per Quality-adjusted Life Year (QALY). Almost 52 hypoglycaemic events per patient were avoided with the use of insulin glargine compared to NPH, at an incremental lifetime cost of 7,140 TL per patient. The cost-effectiveness of insulin glargine is reduced when modelling only those benefits considered in the trial setting, while the cost-effectiveness profile can be expected to further improve in patients with higher HbA1c levels at baseline.

**Conclusion:** It is difficult to interpret the results of modelling as there is no official cost-effectiveness threshold in Turkey. However, the results may be evaluated using thresholds derived according to methodology proposed by the World Health Organisation (WHO). Insulin glargine is expected to be costeffective compared to NPH insulin, with an ICER below three times the estimated gross domestic product (GDP) per capita; 56,850 TL.

## INTRODUCTION

The prevalence of diabetes is increasing rapidly worldwide, with lifestyle changes and improved economic conditions contributing factors to the substantial rise in patient numbers. According to recent International Diabetes Federation (IDF) estimates, the total number of people with diabetes will rise from 366 million in 2011 (8.3%) to 552 million by 2030, a prevalence rate of 9.9%.[1] The second Turkish Diabetes Epidemiology Study (TURDEP-II) found a higher prevalence rate of 16.5% in the Turkish population in 2010 (approximately 6.5 million), with an additional 14.5%, 7.9% and 8% prevalence of isolated impaired fasting glucose (IFG), impaired glucose tolerance (IGT) and pre-diabetes.[2] It has previously been estimated that in Turkey, 300,000 individuals develop diabetes each year.[3]

In 2008, diabetes was the cause of death in approximately 2.2% of the Turkish population and the fourth major contributor to health expenses. The Turkish Diabetes Foundation estimated that annual treatment costs varied between approximately €340 in diabetic patients without any complications, to €880 in those with complications.[4]

A stepwise approach is conventionally taken in the management of type 2 diabetes mellitus (T2DM), in which more intensive strategies are required as the condition progresses. If optimal glycaemic control cannot be achieved through lifestyle modifications alone, progression to oral antidiabetic drugs (OADs) and insulin becomes necessary.[5] Good glycaemic control is important in the prevention of chronic complications associated with diabetes,[6] and can be improved by the timely initiation of insulin.[7] The position statement of the American Diabetes Association (ADA) and European Association for the Study of Diabetes (EASD) recommends the addition of insulin therapy to lifestyle intervention strategies and metformin therapy, in patients with a glycated haemoglobin (HbA1c) level exceeding 7.0%.[8] Insulin analogues are being increasingly used in the treatment of diabetes in Turkey.[9] The Society of Endocrinology and Metabolism of Turkey (SEMT) guidelines recommend therapy progression in patients with HbA1c >6.5%. The guidelines include insulin as a second-line therapy option for patients failing to achieve adequate glycaemic control with metformin alone, especially in those with HbA1c >8.5%.[10]

However, in clinical practice the timely initiation of insulin therapy and achievement of glycaemic targets deviates from the recommendations presented in the guidelines.[11] Indeed, the adherence of Turkish clinicians to the SEMT guidelines has been found inadequate, with negative impact on glycaemic control.[12] Delays in the progression of treatment in the management of T2DM can result in extended periods of poor glycaemic control, which ultimately increase the risk of micro- and macro- vascular complications.[13, 14] Despite the efficacy of insulin therapy, concerns regarding the administration of injections, hypoglycaemia and weight gain[15] fuel physician and patient reluctance to initiate insulin therapy. Hypoglycaemic events can be traumatic experiences with a substantial impact on quality of life.[16] Furthermore, retrospective analysis of the Action to Control Cardiovascular Risk in Diabetes (ACCORD) trial study has found that symptomatic, severe hypoglycaemia is associated with an increased risk of mortality.[17] Thus, there is a recognised trade-off between glycaemic control and hypoglycaemia risk.

There are still uncertainties regarding the optimal insulin treatment regimens for T2DM, but the long-acting insulin analogues seem promising. Patients failing on OADs have been shown to benefit from lower rates of hypoglycaemia and less weight gain when therapy with insulin glargine is initiated in early T2DM, compared with patients initiated on insulin therapy at later stages of the disease.[18] A large body of clinical trial data has demonstrated the advantages of insulin glargine therapy in combination with OADs in patients with T2DM, compared with neutral protein Hagedorn (NPH) insulin.[19] The intermediate-acting insulin, NPH insulin, has traditionally been used for insulin introduction in patients with T2DM. Many studies have found that patients treated with insulin glargine achieved similar HbA1c levels to those treated with NPH, but with a lower rate of hypoglycaemia in T2DM,[19,20,21] while one trial reported an improvement in HbA1c.[22]

This study aims to assess the cost-effectiveness of T2DM treatment with insulin glargine compared to NPH insulin in Turkey, from a third party payer's perspective.

## METHODS

### Model

The economic model presented in this paper was based upon the model evaluated by the National Institute for Health and Clinical Excellence (NICE) in 2002 and has been described in detail elsewhere.[23] In summary, the model is a discrete event simulation (DES) in which the progression of T2DM can be modelled for a cohort of up to 1,000 subjects, using annual time increments over the modelled time horizon of up to 40 years (Figure 1).

**Figure 1.** Flow Diagram of the T2DM Discrete Event Simulation Model

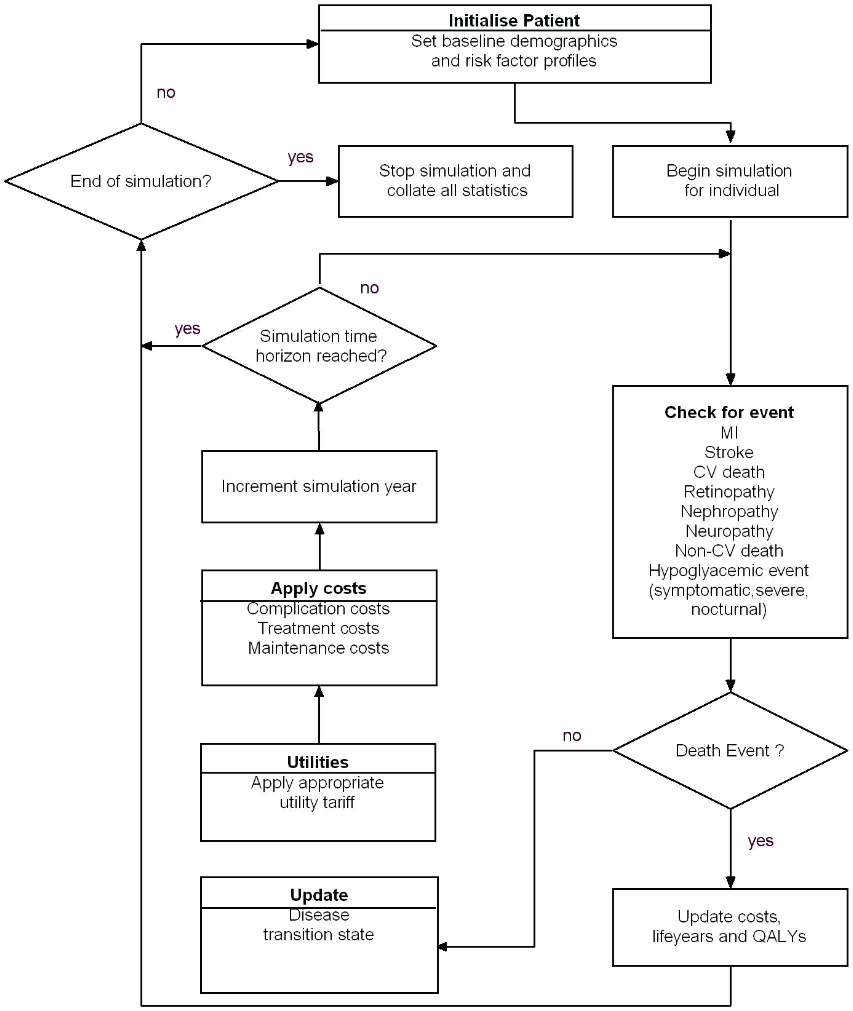

MI=myocardial infarction; CV=cardiovascular; QALY=quality-adjusted life year (score)

Modelling micro- and macro- vascular events in T2DM has been undertaken by encoding the UK Prospective Diabetes Study (UKPDS) 68[24] equations for the prediction of diabetes-related complications; a series of Weibull proportional hazards regression equations. The model calculates the annual number of nocturnal, symptomatic and severe hypoglycaemic events expected and incorporates non-cardiovascular mortality via Turkish gender-specific life tables.

As the simulation progresses, the values of patient risk factors change thereby altering the likelihood of event occurrence over time. Possible treatment effects modelled are the improvement in glycaemic control and reduction in hypoglycaemic events.

### Data Inputs

The mean demographic and baseline risk factor profile utilised in the base case analysis was based on the Turkish results for all T2DM patients in the International Diabetes Management Practice (IDMPS) study[25] (Table 1).

**Table 1.** Baseline Characteristics of T2DM Patients in Turkey for the Base Case, Reported by Gordon et al[27]

| Variable | Base Case | Gordon et al. |
|---|---|---|
| Age (years) | 56.52 | 60.8 |
| Gender (% female) | 57.0 | 45.6 |
| Duration with diabetes (years) | 8.19 | 6.2 |
| Weight (kg) | 80.74 | 85.3 |
| Height (metres) | 1.64 | Not reported |
| Total cholesterol (mmol/L) | 5.11 | Not reported |
| HDL cholesterol (mmol/L) | 1.29 | Not reported |
| Systolic blood pressure (mm Hg) | 129.51 | Not reported |
| HbA1c (%) | 7.72 | 9.5 |
| Smoking (%) | 14.00 | Not reported |

### Glycaemic Control

Following its positive evaluation by NICE in 2002, glargine became an established basal insulin in the United Kingdom[26] enabling the evaluation of glycaemic control in a large number of patients in a general clinical setting.

Model inputs have been informed by results of an analysis of UK practice patterns and patient outcomes data from The Health Improvement Network (THIN) database presented by Gordon *et al.*[27] An in-depth comparison of patients newly prescribed insulin glargine, NPH, detemir, and premix insulin was conducted and multiple regression analysis used to model the primary outcome; change in HbA1c at 12 months. After adjustment for significant and clinical covariates, the reported reduction in HbA1c associated with glargine compared to NPH was 0.19%. This value was applied in the base case analysis.

Total daily insulin dosages observed in the THIN database were lowest with glargine (basal/total: 0.56/0.66 U/kg/day) and highest with NPH (basal/total: 0.64/0.81 U/kg/day). After adjusting for this difference in daily dosages, a larger difference in glycaemic control was found between glargine and NPH. Further subgroup analysis revealed greater improvement in glycaemic control in patients with a baseline HbA1c of at least 8% and of at least 10% (Table 2).

**Table 2.** Improvement in Glycaemic Control (HbA1c) at 12 months† for Glargine versus NPH; before and after Dose Adjustments

| | HbA1c Reduction† at 12 months | Dose-adjusted HbA1c Reduction at 12 months |
|---|---|---|
| Overall | 0.19 | 0.30 |
| Baseline HbA1c ≥8% | 0.25 | 0.41 |
| Baseline HbA1c ≥10% | 0.27 | 0.47 |

HbA1c=glycated hemoglobin; †HbA1c reduction adjusted for age, sex, duration of diabetes, weight, number of oral therapies and comorbidities

## Relationship between HbA1c and Hypoglycaemia

The model incorporates the annual probability of experiencing a severe hypoglycaemic event from the Diabetes Control and Complications Trial (DCCT),[13] in addition to annual rates of nocturnal and symptomatic hypoglycaemic events sourced from Riddle *et al.*[19,28] (Table 3). There is a recognised correlation between glycaemic control and the risk of hypoglycaemia and the nature of this relationship between HbA1c and hypoglycaemia has been characterised as shown in Figure 2.

**Table 3.** Estimated Annual Hypoglycaemia Risks and Number of Expected Events

| Hypoglycaemia | Value | Source |
|---|---|---|
| Risk of Severe | †0.462 | DCCT,[13] Leese *et al.*[34] |
| Number of Nocturnal | 4.9 | Riddle *et al.*[19] |
| Number of Symptomatic | 16.8 | Riddle *et al.*[28] |

†predicted reduction for an equivalent risk of symptomatic hypoglycaemic episodes

**Figure 2.** Rate of Severe Hypoglycaemia in Patients Receiving Intensive Therapy; According to their Mean Glycosylated Haemoglobin Values during the DCCT Trial[13]

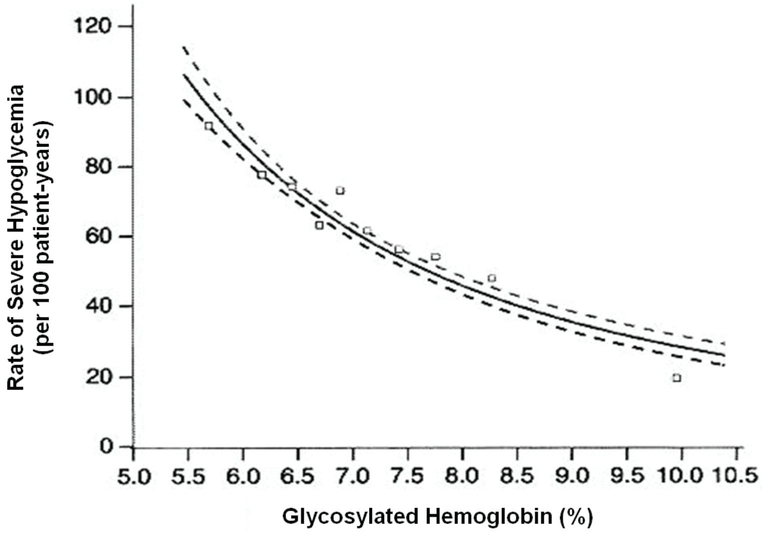

The reductions in hypoglycaemic events associated with glargine compared to NPH were taken from Mullins *et al.*[29] (10%, 35%, and 40% for symptomatic, nocturnal, and severe events respectively) and calibration coefficients were applied to the Mullins equations to automatically derive baseline hypoglycaemia rates dependent on HbA1c levels at baseline (Figure 3).

**Figure 3.** Illustrating Re-calibration of the Mullins Meta-regression Equations; for Setting HbA1c-specific Levels of Baseline Hypoglycaemia

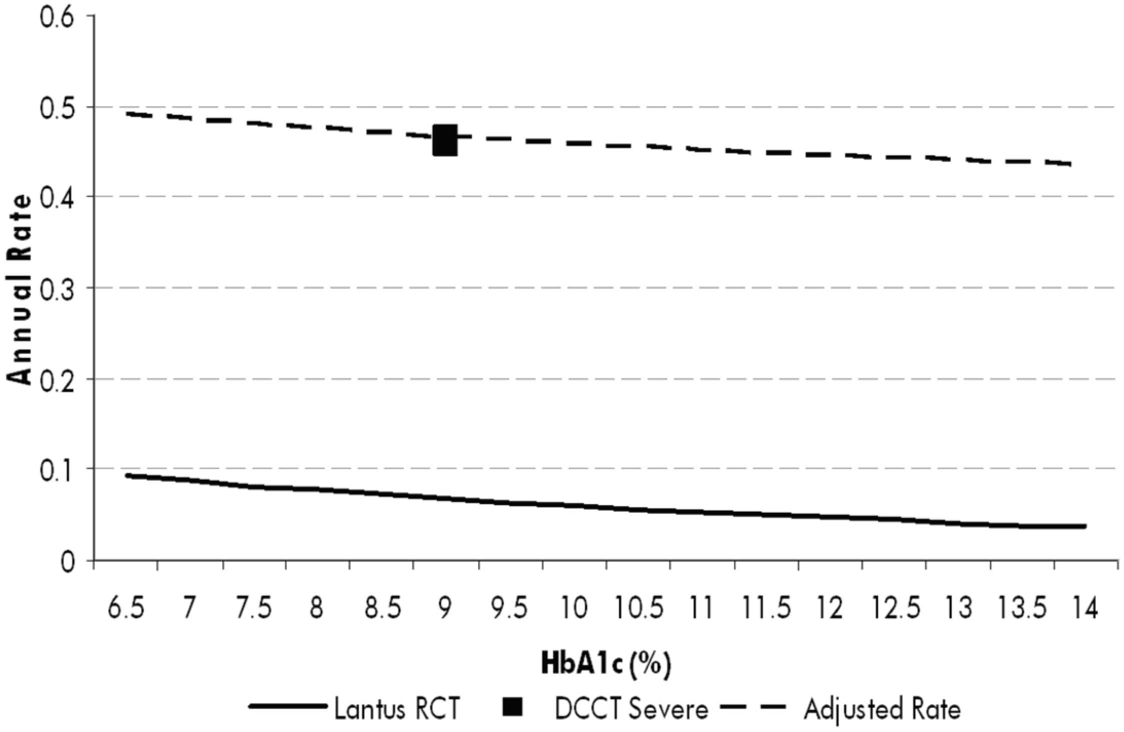

RCT=randomised control trial; DCCT=Diabetes Control and Complications Trial; HbA1c=glycated hemoglobin

### Costs

All costs applied in the model are presented in 2012 Turkish Lira (TL), adjusted for inflation using International Monetay Fund (IMF) price indices for Turkey where required.[30]

The official price list was used to inform unit costs of insulin glargine and NPH.[31] In the base case analysis, treatment costs were applied according to the daily insulin dosages reported in Gordon *et al.*[27] The costs applied to diabetes-related complications were derived via the opinions of a panel of Turkish experts, specialising in the fields of internal diseases/endocrinology, cardiology, eye diseases, neurology, orthopaedics/general surgery, and urology (Table 4).

**Table 4.** Fatal and Non-fatal Event and Maintenance Costs Applied in the T2DM Model

| | Costs (TL) | | |
|---|---|---|---|
| **Complication** | **Fatal** | **Non-fatal** | **Maintenance** |
| **Macrovascular** | | | |
| Ischaemic Heart Disease | 3129.33 | 1851.06 | 727.04 |
| Myocardial Infarction | 3322.95 | 2022.35 | 684.56 |
| Congestive Heart Failure | 9717.60 | 1239.68 | 930.59 |
| Stroke | 929.00 | 996.25 | 1061.37 |
| **Microvascular** | | | |
| Retinopathy: Blindness | - | 956.82 | 740.29 |
| Renal: Dialysis | - | 27 055.72 | 27 055.72 |
| Amputation | 2159.96 | 2159.96 | 4582.44 |
| Severe Hypogiyacaemia | 74.64 | 74.64 | 0.00 |

TL=Turkish Lira

Either fatal or non-fatal event costs are applied in the year of incidence, with annual maintenance costs applied in all subsequent years for non-fatal events. Maintenance costs associated with ischaemic heart disease and myocardial infarction could not be sourced; hence they were estimated by adjusting the cost of congestive heart failure maintenance according to the ratio observed in the United Kingdom.

As subjects are assumed to incur severe vision loss or blindness in both eyes simultaneously, the blindness event can occur only once. Dialysis costs are annual weighted mean costs for peritoneal and haemodialysis. A cost is applied to severe hypoglycaemic events only.

### Health-related Utility

Decrements in health-related utility associated with diabetes-related complications were derived from the UKPDS 62 study[32] or generated via the Health Outcomes Data Repository (HODaR) database.[33] The HODaR project collected patient data characterising demography, productivity, self-reported risk factors, and health-related quality of life (HRQoL) using the SF-36 and EQ-5D from patients discharged from the Cardiff and Vale NHS Trust, which treats the local population (n=424,000) and acts as a tertiary centre for the rest of Wales (UK). Data were linked anonymously to routinely collected inpatient and outpatient data. Patients were excluded from the analysis if they were under age 18 years, discharged with a primary diagnosis relating to a psychiatric illness or had completed a survey in the previous 6 months. All subsequent events are assumed to incur the same utility decrement as in the primary event (Table 5).

**Table 5.** Health-related Utility Decrements (EQ-5D) and Associated End Points

| Event | Utility Decrement | | Source |
|---|---|---|---|
| | Primary | Subsequent | |
| **Macrovascular** | | | |
| Ischemic Heart Disease | 0.090 | 0.090 | UKPDS 62[32] |
| Myocardial Infarction | 0.066 | 0.066 | HODaR Survey[47] |
| Congestive Heart Failure | 0.108 | 0.108 | UKPDS 62 |
| Stroke | 0.114 | 0.114 | HODaR Survey |
| **Microvascular** | | | |
| Pre-blind | 0.029 | 0.029 | HODaR Survey |
| Blind | 0.074 | 0.074 | UKPDS 62 |
| End-state Renal Disease | 0.263 | 0.263 | UKPDS 62 |
| Dialysis | 0.305 | 0.305 | HODaR Survey |
| Transplant | 0.075 | 0.075 | HODaR Survey |
| Amputation | 0.280 | 0.280 | UKPDS 62 |

UKPDS=United Kingdom Prospective Diabetes Study;
HODaR=Health Outcomes Data Repository

The utility associated with hypoglycaemic events was modelled via multivariate regression models, relating the fear of hypoglycaemia to changes in health-related utility.[16] The equations developed used pooled data from two postal surveys conducted in Cardiff, United Kingdom (n=1,305 responses), in which the fear of hypoglycaemia was characterised using the fear of hypoglycaemia survey (FHS [eight question worry sub-scale only]) and health-related utility using the EQ-5D index.

The analysis revealed the FHS value to be the best estimate of the EQ-5D, while the number of hypoglycaemic events was found to be an important predictor of the FHS value. A two-stage approach was therefore adopted to predict EQ-5D; the relationship between frequency of hypoglycaemic events and FHS value was estimated, before estimating the EQ-5D using the predicted FHS value. Validation exercises proved the predictive power of the equations to be strong, with actual and predicted FHS score and EQ-5D values closely matched across all hypoglycaemia frequency and severity categories and across quartiles of the FHS.

Costs and benefits, measured in terms of Quality-Adjusted Life Years (QALYs), were assessed over a 40-year horizon and discounted at 3.5%.

## RESULTS

### Base Case

The cost per QALY associated with insulin glargine treatment compared to insulin NPH was estimated at 40,101 TL under base case conditions. Per person, the lifetime difference in costs was estimated to be 7,140 TL, with an estimated benefit of 0.178 QALYs and a total of 52 hypoglycaemic events avoided. This translates to a cost of 137 TL per hypoglycaemic event avoided (Table 6).

**Table 6.** Lifetime Results for a Cohort of 1,000 Patients Comparing Insulin Glargine to NPH Insulin Use

| Scenario | Lifetime Difference | | | Costs (TL) per Hypoglycaemic Event Avoided | Costs (TL) per QALY |
|---|---|---|---|---|---|
| | Total Costs (TL) | Total QALYs | Hypoglycaemic Events* | | |
| **Base Case** | **7 139 980** | **178** | **-51 976** | **137** | **40 101** |
| Gordon Demographics | 6 350 526 | 159 | -39 374 | 161 | 40 025 |
| No HbA1c Benefit | 7 181 653 | 144 | -53 394 | 134 | 49 916 |
| **Dose-adjusted HbA1c Reduction** | | | | | |
| Overall | 8 403 624 | 200 | -50 369 | 167 | 42 071 |
| Baseline HbA1c ≥8% | 8 622 668 | 220 | -49 244 | 169 | 37 756 |
| Baseline HbA1c ≥10% | 8 214 494 | 261 | -72 448 | 113 | 31 418 |

*Sum of all hypoglycaemic events (symptomatic, nocturnal and severe);
HbA1c=glycated heboglobin;
TL=Turkish Lira;
QALY=quality-adjusted life years

## Supplementary Analyses

Further analysis implementing the demographic profile presented by Gordon *et al.*[27] demonstrated only minor changes in the ICER value compared to the base case. Differences in mean age, gender, duration of diabetes, weight, and HbA1c at baseline resulted in a similar ICER of 40,025 TL.

Analysis evaluating the cost-effectiveness of glargine without improvement in glycaemic control associated with glargine resulted in an increased ICER. If no account is taken of the HbA1c benefit associated with glargine, no differences in the expected rates of diabetes-related conditions are expected between glargine and NPH. However, patients receiving glargine still experience clinical benefit in the form of fewer hypoglycaemic events than those on NPH.

Dose-adjusted improvements in glycaemic control were implemented in further analyses using therapy costs based on the weighted mean of dosages reported in Gordon *et al.*[27] The mean HbA1c values for each subgroup at baseline were estimated from the reported risk factor profile; 10.0% and 11.1% for subgroups with baseline ≥8% and ≥10% respectively.

As HbA1c is a risk factor for cardiovascular disease, patients with improved glycaemic control are expected to experience fewer cardiovascular events. As the modelled reduction in HbA1c associated with glargine treatment increased for patients with a higher HbA1c level at baseline, the number of diabetes-related events avoided by these patients also increased. A greater number of diabetes-related events avoided due to glargine treatment compared to NPH, lead to a larger difference in QALYs and smaller difference in lifetime costs between the two insulins, and consequently improved cost-effectiveness results in these subsets of patients.

## Univariate Sensitivity Analysis

One way sensitivity analysis on key model parameters showed the results to be most sensitive to large changes to the effectiveness of glargine with respect to hypoglycaemic events and to all costs (Table 7).

**Table 7.** Sensitivity Analysis on Key Model Parameters for Glargine versus NPH

| Glargine vs. NPH Scenario | Cost/QALY |
|---|---|
| **Base Case** | **40 101** |
| Age - 10 years | 42 883 |
| Age + 10 years | 40 628 |
| Weight - 10 kg | 35 460 |
| Weight + 10 kg | 43 772 |
| Time Horizon | |
| 5 years | 59 289 |
| 10 years | 50 484 |
| 20 years | 45 213 |
| 30 years | 40 981 |
| Discount Rate | |
| 0% | 36 367 |
| 0% Benefits/5% Costs | 19 909 |
| 3% Benefits/3% Costs | 39 581 |
| 7% Benefits/7% Costs | 43 544 |
| Utility Values | |
| 50% Reduction of First year Decrement | 40 177 |
| 50% Reduction in Subsequent Year Decrements | 40 714 |
| Treatment Effects | |
| 50% Reduction in Hypoglycaemia Efficacy | 67 094 |
| Severe Hypoglycaemia Risk Eduction = 0% | 83 919 |
| Event Costs | |
| 50% Reduction of All Event Costs | 40 721 |
| 50% Increase of All Event Costs | 39 482 |
| All Costs (Events and Treatment) | |
| 50% Reduction of All Costs | 20 051 |
| 50% Increase of All Costs | 60 152 |

NPH=neutral protamine Hagedorn; QUALY=quality adjusted life years

Varying the input values for age, weight and discount rate had only a minor effect on the overall result across all scenarios modelled. Similarly, reductions to the utility decrements associated with diabetes-related events had only a minor effect on the modelled outcome. Discounted costs (5%) but not benefits resulted in relatively higher benefit and thus a decreased ICER (19,909 TL).

Reducing the time horizon led to an increased ICER, as the long-term benefit of fewer diabetes-related complications associated with glargine were not fully accounted for. The 50% reduction in insulin glargine's hypoglycaemia efficacy had a negative effect on the model's results, with an increased ICER of 67,094 TL for glargine versus NPH. Setting the reduction of severe hypoglycaemia risk to zero percent had a greater impact on results, with an ICER of 83,919 TL versus NPH.

A reduction to all event costs in the model decreased the ICERs significantly; likewise an increase in all event and treatment costs increased the ICERs significantly. Indeed, the upper stored cost profile resulted in an ICER of 60,152 TL versus NPH.

## DISCUSSION

It is generally thought that severe hypoglycaemia is not as great a concern in patients with T2DM as in T1DM; however Leese *et al.*[34] demonstrated the risk of experiencing a severe event requiring hospitalization was as important for T2DM patients receiving insulin as for T1DM patients.

Differential patterns of hypoglycaemia exist amongst different study or population types. For instance, randomised controlled trials (RCTs) have been shown to demonstrate lower levels of hypoglycaemia than observational studies as part of the 2006 Australian evaluation of insulin glargine presented to the Pharmaceutical Benefits Advisory Committee (PBAC).[35] These differing event rates may be commensurate with outcomes from trials of intensified insulin therapy (IIT), which demonstrated that successfully achieving glycaemic control is associated with an increased burden of hypoglycaemia. Indeed the risk of severe hypoglycaemic events increased up to threefold over 'conventional' therapy.[13, 36]

Despite the failure of conventional meta analyses[37,38] of RCT results to identify clinically meaningful benefit of glargine over NPH, several randomised trials[21,39,40,41,42] and well-specified observational studies[43] report up to a 66% rate reduction[40] in all types of hypoglycaemic events in conjunction with a reduction in HbA1c of up to 0.5%. Furthermore, meta-regression analysis has successfully demonstrated the effect of glargine on the relationship between HbA1c and hypoglycaemia. Mullins *et al.*[29] defined this relationship using pooled data from all phase III to IV clinical trials comparing glargine and NPH.

Insulin glargine produces clinical benefits for patients with T2DM through the reduction of both HbA1c levels and hypoglycaemic events. The lifetime risk of macro- and micro-vascular events is reduced as a result of improved glycaemic control, leading to long-term life expectancy and quality of life gains. Further improvements in quality of life are achieved through the avoidance of hypoglycaemic events, which may also have positive implications for the timely initiation of insulin therapy, patient compliance and persistence of therapy.

It is difficult to interpret the results of the cost-effectiveness analysis because there is no official cost-effectiveness threshold in Turkey, since cost utility analyses are not considered valid for the reimbursement of pharmaceuticals. However, the results of the present analysis can be usefully analysed using thresholds calculated using methodology from the World Health Organization (WHO), as follows:

Therapy is very cost-effective if the ICER is less than GDP per capita (app. 18,950 TL), is cost-effective if the ICER is between one and three times GDP per capita (app. 18,950 - 56,850 TL) and is not cost-effective if the ICER is greater than three times GDP per capita (app. 56,850 TL) (GDP per capita estimated by IMF).[30]

More generally, in European cost-effectiveness studies as a whole, it is generally agreed that an intervention that provides one extra year of quality-adjusted life at a cost of €30,000 provides good value, although explicit cost-effectiveness thresholds are rarely stated.[44] This equates to almost 69,500 TL (2012 average exchange rate: €1 = 2.3135 TL).[45]

Insulin glargine can thus be considered cost-effective compared to NPH under base case conditions, as the ICER of 40,101 TL was well below the cost-effectiveness threshold typically applied in Europe, or derived using WHO methodology. Insulin glargine can also be considered cost-effective under all scenarios modelled as part of supplementary analysis and the majority of univariate sensitivity analyses conducted.

An increase of all costs (event and treatment) by 50% or reduction in the efficacy of glargine with respect to hypoglycaemia by 50%, result in ICER values within the range typically accepted as cost-effective in Europe, however in excess of the threshold derived according to WHO methodology. Reducing the effect of glargine on severe hypoglycaemia to zero was the only tested scenario under which glargine cannot be considered cost-effective compared to NPH, as the ICER exceeds both the thresholds corresponding to 3*GDP and typically applied in Europe.

Weight is known to have significant implications for quality of life, mortality and health related costs.[46] However, the model does not simulate changes in patient weight as a result of either natural progression or initiation of insulin therapy. Weight is a recognised risk factor for cardiovascular events and indeed is included in the UKPDS 68[24] equation here used to predict the incidence of congestive heart failure. Furthermore, fears regarding weight gain as a result of treatment have been shown to be a significant factor inhibiting medication adherence in T2DM patients.[15]

A number of studies have reported comparable[19,21] or smaller[20] weight gains associated with insulin glargine compared to NPH. Comparisons made by Gordon *et al.*[27] showed that while patients on all assessed regimens generally gained weight over the first 12 months, the increase in weight associated with insulin glargine was smaller than with NPH (mean 1.9 kg with glargine vs. 2.3 kg with NPH). Thus the inclusion of weight changes at initiation of insulin would not be expected to result unfavourably on the cost-effectiveness results reported in this analysis.

Where data are available, inputs specific to the Turkish population have been utilised, however this does not extend to the HbA1c efficacy data obtained from the United Kingdom. The management of T2DM in Turkey is likely to be similar to that of the United Kingdom, since clinicians follow an incremental approach to therapy, often refer to international guidelines and will encounter similar barriers to therapy progression. The use of observational data from clinical practice is particularly useful in view of the recognised disparity between guidelines and clinical practice.

## CONCLUSION

Results of the modelling conducted suggest that insulin glargine may be considered cost-effective compared to NPH in a Turkish setting, based on methodology proposed by WHO and also the threshold typically applied in Europe. Insulin glargine is expected to be most cost-effective in patients with higher HbA1c levels at baseline and to remain cost-effective compared to NPH, even when the HbA1c benefits associated with glargine are not accounted for.

## ACKNOWLEDGEMENTS

Phil McEwan and colleagues developed the model used in this analysis and Richa Goyal Rai conducted background research contributing to the manuscript's introduction.

### Conflict of Interest Declaration

The study was supported by Sanofi Turkey, by whom T. Sahin and D. Ozkaya are employed. H. Bennett acted as consultant to Sanofi Turkey.
